# Protective potential of frankincense essential oil and its loaded solid lipid nanoparticles against UVB-induced photodamage in rats via MAPK and PI3K/AKT signaling pathways; A promising anti-aging therapy

**DOI:** 10.1371/journal.pone.0294067

**Published:** 2023-12-21

**Authors:** Eman A. Kotb, Riham A. El-Shiekh, Wessam H. Abd-Elsalam, Nesrine Salah El Dine El Sayed, Nebal El Tanbouly, Amira Safwat El Senousy

**Affiliations:** 1 Department of Pharmacognosy, Faculty of Pharmacy, Cairo University, Cairo, Egypt; 2 Department of Pharmaceutics and Industrial Pharmacy, Faculty of Pharmacy, Cairo University, Cairo, Egypt; 3 Department of Pharmacology and Toxicology, Faculty of Pharmacy, Cairo University, Cairo, Egypt; University of the Witwatersrand, SOUTH AFRICA

## Abstract

Frankincense oil has gained increased popularity in skin care, yet its anti-aging effect remains unclear. The current study aimed to investigate the anti-photoaging effect of frankincense (*Boswellia papyrifera* (Del.) Hochst., Family Burseraceae) essential oil in an *in vivo* model. The oil was initially extracted by two methods: hydro-distillation (HD) and microwave-assisted hydro-distillation (MAHD). GC/MS analysis revealed the dominance of *n*-octyl acetate, along with other marker compounds of *B*. *papyrifera* including octanol and diterpene components (verticilla 4(20) 7, 11-triene and incensole acetate). Thereafter, preliminary investigation of the anti-collagenase and anti-elastase activities of the extracted oils revealed the superior anti-aging effect of HD-extracted oil (FO), comparable to epigallocatechin gallate. FO was subsequently formulated into solid lipid nanoparticles (FO-SLNs) *via* high shear homogenization to improve its solubility and skin penetration characteristics prior to *in vivo* testing. The optimimal formulation prepared with 0.5% FO, and 4% Tween^®^ 80, demonstrated nanosized spherical particles with high entrapment efficiency percentage and sustained release for 8 hours. The anti-photoaging effect of FO and FO-SLNs was then evaluated in UVB-irradiated hairless rats, compared to Vitamin A palmitate as a positive standard. FO and FO-SLNs restored the antioxidant capacity (SOD and CAT) and prohibited inflammatory markers (IL6, NFκB p65) in UVB-irradiated rats *via* downregulation of MAPK (pERK, pJNK, and pp38) and PI3K/AKT signaling pathways, alongside upregulating TGF-β expression. Subsequently, our treatments induced Procollagen I synthesis and downregulation of MMPs (MMP1, MMP9), where FO-SLNs exhibited superior anti-photoaging effect, compared to FO and Vitamin A, highlighting the use of SLNs as a promising nanocarrier for FO. In particular, FO-SLNs revealed normal epidermal and dermal histological structures, protected against UVβ-induced epidermal thickness and dermal collagen degradation. Our results indicated the potential use of FO-SLNs as a promising topical anti-aging therapy.

## Introduction

Frankincense (olibanum), an oleogum resin obtained from *Boswellia* trees (Family Burseraceae), consists of 5–9% essential oil (mono-, di- and sesquiterpenes, alkyl esters and alcohols), 65–85% alcohol soluble resins (di- and triterpenes) and the remaining water-soluble gums (polysaccharides) [1, 2].

The genus *Boswellia* comprises more than 20 species and is distributed across Northeast Africa, Southern Arabia and India [3–5], with mainly four commercially relevant species, namely *B*. *serrata* Roxb. ex Colebr., *B*. *sacra* Flueck. (syn. *B*. *carterii*), *B*. *papyrifera* (Del.) Hochst. and *B*. *frereana* Birdw [2, 6–8]. Indian olibanum is obtained from *B*. *serrata*, while Sudanese olibanum is derived from *B*. *papyrifera*, which occurs in Sudan, Ethiopia, and some East African regions [3–5]. *B*. *sacra* mainly grows in the Middle East and the Arabian Peninsula, whereas B. *frereana* occurs in NorthEast Africa (Ethiopia and Somalia) [7, 9].

Frankincense has been extensively used for medical purposes, for thousands of years, in traditional Islamic, Chinese and Indian medicine [6, 10], in addition to its use as chewing gum, incense or in perfumery [11]. Frankincense products are available as resins, extracts and essential oils. Various frankincense preparations are marketed as dietary supplements as well as their wide use as valuable ingredients in skin care products [7, 11]. Pharmacological research highlighted myriad biological activities of frankincense essential oil (FEO) including antimicrobial, anti-inflammatory, analgesic, anticancer and immunomodulatory activities [6, 10, 12–15].

Recently, more attention has been paid to the topical effects of frankincense. Topical application of FEO and its active ingredients (α-pinene, linalool, 1-octanol) exhibited significant anti-inflammatory and analgesic effects through inhibiting induced inflammatory infiltrates and COX-2 overexpression in xylene-induced ear edema model [13]. Its anti-inflammatory effect has been demonstrated *via* inhibiting the transactivation of NF-kB through the stabilization of I_k_Bα [16]. Besides, the oil possessed a promising potential to modulate the biological processes of inflammation and tissue remodeling in human skin [10]. Additionally, FEO-loaded hydrogel wound dressings demonstrated antimicrobial and wound-healing efficacy in rats [17].

More than three hundred volatiles have been reported in FEO, showing broad variation among different species [1, 8]. However, predominant constituents were identified in the essential oil of different *Boswellia* species as characteristic of these species [2]. In this context, the main constituents characterizing the most important commercial sources of frankincense viz., *B*. *serrata*, *B*. *sacra*, *B*. *papyrifera* and *B*. *frereana* are α-thujene, α-pinene, *n*-octyl acetate and p-cymene, respectively [2].

Hydro-distillation and steam distillation are the most commonly adopted methods for essential oil extraction in the laboratory and at the industrial scale [18]. Microwave-assisted hydro-distillation (MAHD) has been developed among other novel techniques for extracting essential oil, being particularly effective [18–20]. It integrates rapid microwave heating with traditional solvent extraction, thus accelerating essential oil extraction together with improving its yield and quality [21]. MAHD of frankincense (*B*. *serrata*) essential oil has been previously reported, showing enhanced contents of its main components (α-thujene and α-pinene) [22].

Skin aging is attributed to the cumulative effects of intrinsic and extrinsic factors. Intrinsic aging occurs naturally with age, while extrinsic aging is elicited by environmental factors, in particular UV radiation [23]. Around 80% of facial skin aging is credited to UV exposure [24]. Safeguarding against excessive UVB-induced damage is beneficial in the prevention of cancer, aging and other human illnesses [25, 26]. UV radiation induces accumulation of ROS in the skin, which in turn activates the MAPK pathway and inflammatory cytokines, stimulating MMPs synthesis and leading to degradation of the extracellular matrix components such as collagen and elastin, thus contributing to wrinkle formation, one of the most distinguished hallmarks of photo-damaged skin [27, 28]. Photo-aged skin is characterized by roughness, dryness, irregular pigmentation, loss of elasticity and deep wrinkles [27]. Recently, the rising demand for beauty and youth coupled with the banning of using traditional chemicals enhanced the quota of “natural cosmetics” over synthetic ones in the market [29]. Accordingly, exploring the cosmetic potential of more plants is in need. Lots of studies proved the potential of herbal products for skin care purposes due to their anti-inflammatory, moisturizing and UV-protective, and antioxidant effects [30, 31].

In recent years, cosmeceuticals like retinoids, antioxidants (alpha-tocopherol and ascorbic acid) and herbal ingredients have been formulated as nanosized carriers such as vesicular systems, lipidic nanoparticles and nanoemulsions. These nanosized delivery systems bear many advantages over the conventional preparation; namely, boosted dispersibility, stability and hence efficacy [32–34]. Considering the importance of essential oils as sources of bioactive molecules for the cosmetic industry, researchers investigated the use of nano lipid forms of some essential oils as new delivery systems *in vitro* and *in vivo* for the treatment of skin disorders [35], due to dehydration, melanoma, inflammation, wounding, and fungal infections. Data reported that lipid nano-systems allow a deep interaction between lipid matrix and skin strata allowing the easy and prolonged release and efficacy of loaded essential oils [36]. In this aspect, the use of solid lipid nanoparticles (SLNs) has been proposed for the cutaneous application of essential oils to improve the hydration and elasticity of the skin [37].

Most of the previous studies clinically proved the anti-aging effect of *Bosewellia serrata* resin extracts and its main components; the pentacyclic triterpene boswellic acids [38, 39]. Despite the increasing interest in frankincense oil as one of the most important commercial essential oils available in the international market, becoming increasingly popular for promoting skin health [15, 40], its anti-aging effect is still unclear. To add, the essential oil of *Boswellia papyrifera* has only been recently examined despite being one of the most widely propagated species in Africa.

In this study, the volatile composition of FEO obtained by HD and MAHD was analyzed and compared regarding their *in vitro* anti-collagenase and anti-elastase activities. Thereafter, a nanosystem had been designed for the more active hydro-distilled essential oil to formulate a suitable delivery system for frankincense oil. The protective effect of hydro-distilled FEO against ultraviolet B-irradiated hairless rats was then evaluated by assessing its antioxidant, anti-inflammatory and anti-aging potential, *via* incorporation into solid lipid nanoparticles to improve its topical efficacy.

## Materials and methods

### Plant material and extraction of frankincense essential oil

Frankincense (oleogum resin of *Boswellia papyrifera* (Del.) Hochst., Family Burseraceae, imported from Sudan) was purchased from the local market of herbs and spices in Egypt (Ahmed Abdelrahman EL Harazz) and identified by Dr. Abd El Haleem Abd El Magly (Agriculture Research Center, Department of Fluorescence Research and Plant Classification). A voucher specimen (No 24.02.2022 I) was kept at the Pharmacognosy Department Herbarium, Faculty of Pharmacy, Cairo University. Frankincense essential oil was extracted using two methods: Hydro-distillation (HD) and Microwave-Assisted Hydro-distillation (MAHD) methods.

### Hydro-distillation method

Powdered frankincense (100 gm) was subjected to hydro-distillation for 4 hr (until no more essential oil was obtained) using a Clevenger-type apparatus. The essential oil was extracted with 500 mL of distilled water in a 1 L flask. The system was operated at constant power and under atmospheric pressure. After this time, the extracted essential oil was collected and dried over anhydrous sodium sulfate, weighed and stored at 4°C.

### Microwave-assisted hydro-distillation

MAHD was applied by using a focused microwave apparatus (MARS 240/50, no.907511; CEM Corporation, Matthews, North Carolina, USA), operating at 2450 MHz with a maximum power of 1600W. Powdered frankincense (100 gm) was placed in a 500 mL round-bottomed flask, connected to a Clevenger-type apparatus outside of a microwave oven. The extraction was carried out at 800W power for 60 mins and the temperature was set at 100°C. The oil was collected and dried over anhydrous sodium sulfate, weighed, and stored at 4°C.

### GC-MS analysis of frankincense essential oil

The GC-MS analysis of the frankincense sample was performed using a TRACE GC Ultra Gas Chromatographs (THERMO Scientific Corp., USA), coupled with a thermo mass spectrometer detector (ISQ Single Quadrupole Mass Spectrometer). The GC-MS system was equipped with a Tr-5MS column (30 m x 0.32 mm i.d., 0.25 μm film thickness). Analyses were executed using helium as carrier gas at a flow rate of 1.0 mL/min and a split ratio of 1:10 applying the subsequent temperature program: 60°C for 2 min; increasing at 4.0°C /min to 240°C and held for 5 min. The injector and detector temperatures were set at 210°C. 1 μL of samples (1:10 hexane, v/v) were injected. Mass spectra were obtained by electron ionization (EI) at 70 eV, using a spectral range of m/z 35–500. Components were identified by their retention indices (RI) relative to a series of n-alkanes (C9 -C22) and by matching their mass spectra to the replib, mainlib, wiley and NIST library databases, and literature data [41–43], as well as to those of authentic standards when available.

### *In vitro* anti-aging activity of frankincense essential oil

#### Anti-elastase activity

Working solutions were prepared using samples at concentrations of (1000–7.81 μg/mL). The assay was executed as previously described by [44] using epigallocatechin gallate (EGCG) as the positive control. Porcine pancreatic elastase, elastin as the substrate, and the prepared samples/Standard were separately dissolved in pH8 Tris-HCL buffer. Absorbance was read at 538 nm. The results were finally expressed as IC_50_ values.

#### Anti-collagenase activity

The collagenase inhibition activity of [44] was applied using the enzyme from *Clostridium histolyticum*, epigallocatechin gallate (EGCG) as the positive control and the fluorescence intensity was measured at 485nm and 538nm as the excitation and the emission wavelengths, respectively. The results were finally expressed as IC_50_ values.

### Preparation and characterization of frankincense essential oil-loaded solid lipid nanoparticles (FO-SLNs)

#### Formulation of frankincense oil-loaded solid lipid nanoparticles (FO-SLNs)

Frankincense oil-loaded solid lipid nanoparticles (FO-SLNs) were formulated *via* high shear homogenization [45]. In brief, the oily phase (10% of the formulation) consisted of FO at (0.25, 0.375 and 0.5%, w/w) and Compritol^®^ 888 ATO (1%, w/w) as a stabilizer, while the aqueous phase comprised Tween^®^ 80 at (2, 4 and 6%, w/w) as a surfactant in distilled water. Both oily and aqueous phases were preheated to 80°C before mixing. The oily phase was added dropwise to the aqueous phase under mechanical stirring at 15000 rpm followed by homogenization at 24000 rpm for 10 min using Daihan homogenizer (WiseMix™ HG15A, Daihan Scientific, Seoul, Korea). The resulting o/w dispersion was then subjected to ultrasonication for 10 min (ultrasonic processor, GE130, probe CV18, USA). Following sonication, the resulting dispersion was placed at 4 C for 2 h to form FO-SLNs. The dispersion was then ultracentrifuged at 15000 rpm, 4°c for 1 h to separate the FO-SLNs. The supernatant was then withdrawn with a syrige, and the FO-SLNs were then re-suspended by vortexing for 1 min and centrifuged again. Finally, The supernatant was then withdrawn with a syrige to complete the washing. FO-SLNs were stored in the refrigerator till further use.

#### Experimental design and optimization of FO-SLNs

A 3^2^ full factorial design was utilized to optimize the formulation of FO-SLNs and to evaluate the influence of the formulation variables on the studied responses. Two variables, at three levels each, were set: namely the percentage of FO in the formulation (A) and the percentage of Tween^®^ 80 in the formulation (B). On the other hand, three responses had been adopted to be tracked for the optimization of the studied variables: particle size (PS, Y_1_), zeta potential (ZP, Y_2_), and entrapment efficiency percentage (EE%, Y_3_). The 9 experimental runs were performed in triplicate (n = 3) and were conducted in random order to eliminate bias. The optimal FO-SLNs formulation was elected based on the desirability function (the nearest value to 1), where EE% and ZP (as absolute value) were maximized, while PS was minimized.

### *In vitro* characterization of FO-SLNs

#### Determination of particle size, polydispersity index and zeta potential

The mean particle size (PS, z-average diameter), polydispersity index (PDI), and zeta potential (ZP) of FO-SLNs suspensions were determined *via* dynamic light scattering technique using Malvern^®^ Zetasizer Nano ZS90 (Malvern^®^ Instruments Limited, Worcestershire, UK) [46]. All samples were diluted with bidistilled water, and the measurements were performed in triplicate at 25°C, then the values were reported as mean ± Standard deviation [47].

#### Determination of entrapment efficiency percentage (EE%)

The Entrapment efficiency percentage (EE%) of FO in the formulated FO-SLNs was calculated indirectly by determining the content of free FO (unencapsulated in SLNs) in the aqueous surfactant solution. Firstly, 0.3 ml of each formulation was mixed with 1.5 ml of acetonitrile, and then the mixture was subjected to ultracentrifugation (Sigma 3–30 KS, Sigma Laborzentrifugen GmbH, Germany) at 11000 rpm, 4°c for 1 h. The concentration of FO in the supernatant was measured spectrophotometrically at λ max 276 [48].

The percentage of the encapsulated FO in SLNs was calculated using the following equation:

$$EE\%=\frac{Initial\;amount\;of\;FO\;in\;SLNs-Amount\;of\;free\;FO}{Initial\;amount\;of\;FO\;in\;SLNs}\times 100$$ (1)

#### Transmission electron microscopy (TEM)

The shape and structure of the optimal FO-SLNs formulation were scrutinized using a transmission electron microscope (TEM) (JEOL-JEM-2100, Jeol Ltd., Tokyo, Japan). In brief, one drop of the optimal formulation (FO-SLNs) was placed on a copper-coated grid, then subsequently stained with 1% phosphotungstic acid, and finally left to dry at room temperature. The grid was placed in the vacuum chamber of the electron microscope and images were captured at different magnification powers [49].

#### Differential scanning calorimetry analysis (DSC)

The thermotropic properties, phase transition behavior, and crystallinity of the optimal formulation (FO-SLNs) were evaluated using a DSC-60 (Shimadzu, Kyoto, Japan). DSC studies were conducted for FO, Compritol^®^ 888 ATO, a physical mixture of FO and Compritol^®^ 888 ATO, and the optimal FO-SLNs. Accurately 5 mg of each sample were sealed in aluminum pans. The samples were heated in an atmosphere of nitrogen and thermograms were obtained by heating at a constant heating rate of 10°C/min in the range of 20–200°C, and the thermal behavior was integrated *via* an analyzer [50].

#### *In vitro* release study of frankincense oil from SLNs

The dialysis membrane method was used to compare the release of FO from FO-SLNs formulation to that of pure oil. The samples were filled into a dialysis bag (14,000 Daltons) and then introduced into a 50 mL skin-simulated phosphate buffer (pH 6) containing 10% ethanol to maintain sink conditions at 50 rpm for 24 h. The temperature was kept at 32 ± 0.5°C [35]. Samples (2 mL) were withdrawn at 0.5, 1, 2, 4, 6, and 8 h and analyzed spectrophotometrically at λ max 276 nm [48, 51].

#### Stability studies

As per the ICH guidelines, the short term stability studies were executed on three prepared batches of the optimal FO-SLNs. The samples were kept inside amber glass vials at 5 ± 3°C and 25 ± 2°C/60 ± 5% RH for 3 months. At the end of this period, the PS, ZP and EE% were determined and correlated to that attained from recently formulated FO-SLNs. The statistical analysis was performed at *p <* 0.05 using a *t*-test *via* GraphPad Prism Version 9 (San Diego, CA, USA).

### *In vivo* study of the anti-photoaging activity of frankincense essential oil

#### Animals

The experimental study was performed on the hairless skin of adult male Wistar rats weighing 180–220 g (6–8 weeks old), obtained from the animal house of the National Research Center, Cairo, Egypt. Rats were housed in plastic cages and kept in a conditioned atmosphere at 22 ± 3°C and humidity 50–55% with 12 h light/dark cycles, they were fed standard pellet chow (El-Nasr chemical company, Cairo, Egypt) and were allowed free access to water. This study was conducted in accordance with ethical procedures and policies approved by the Institutional Animal Care and Use Committee of Cairo University (CU-IACUC), Cairo University, Egypt, Permit number: CU-III-F- 8–22, which complies with the Guide for the Care and Use for Laboratory Animals published by the US National Institutes of Health (NIH Publication No 85–23, revised 2011).

#### Experimental design

The rats’ dorsal sides were shaved 24 h before starting the experiment. Thirty rats were randomly allocated into five groups, each containing 6 animals (n = 6). Gp I: normal control group; rats were not exposed to any irradiation and did not receive any treatment, Gp II: UVB group; rats were exposed to UVB-irradiation and did not receive any treatment, Gp III: positive standard group (Vitamin A palmitate), Gp IV: FO group and Gp V: FO-SLNs group. Groups III, IV and V were UVB-irradiated and respectively received Vitamin A palmitate, FO and FO-SLNs. A UV lighter (peak emission was 302 nm, CL-1000 M, UVP, Upland, CA, USA) was used for UVB irradiation. UVB irradiation doses were 40–80 mJ/cm^2^ (exposure time was 15–30 s) and the lamp was fixed 5 cm above the platform where rats were placed [52]. Rats received topical treatments once daily on dorsal surfaces, prophylactically 2 h before the UVB exposure for ten consecutive days.

#### Biochemical investigations

The rats were euthanized by cervical dislocation under anesthesia with an overdose of sodium pentobarbital; 60 mg/kg, i.p., afterwards, the treated skin was dismembered out. The homogenized skin tissues were subjected to biochemical examination to explore the antioxidant, anti-inflammatory and anti-aging movement of FO-SLNs *vs*. FO treatment and other supportive groups (normal control, UVB group and positive standard group).

#### Antioxidant activities

The superoxide dismutase reactive substance and the catalase levels were measured using a rat superoxide mutase (SOD) (Catalog No.: MBS036924, California, San Diego (USA)) and catalase (CAT) ELISA kits (Catalog No.: MBS006963, California, San Diego (USA)).

#### Anti-inflammatory activities

Rat IL-6 (Catalog No.: MBS355410, California, San Diego (USA)) and nuclear factor κB p65 (NFκB p65) (Catalog No.: MBS733512, California, San Diego (USA)) were determined by using ELISA kits applying the quantitative sandwich enzyme immunoassay technique.

#### Photo-aging activities

Procollagen I (Catalog No.: LS-F71230, Washington, USA), MMP1 (Catalog No.: LS-F5522, Washington, USA) and MMP9 (Catalog No.: SEA553Ra, Washington, USA) were determined using Procollagen I N-Terminal Propeptide ELISA Kit, ELISA kits 5000 and 2090, respectively.

#### Detection of TGF-β1 in tissue homogenate by real time quantitative PCR (qPCR)

Tissue homogenate was processed for RNA extraction, followed by Reverse Transcriptase (for cDNA synthesis) and quantitative real time PCR. Real-time quantitative polymerase chain reaction (qPCR) differs from regular PCR in including fluorescent reporter molecules in the reaction. These molecules increase proportionally with the increase of DNA amplification in thermocycler.

Total RNA was extracted from tissue homogenate using SV Total RNA Isolation system (Thermo Scientific, USA). This system was performed specifically for transforming growth factor (TGF)-β1. Each sample was determined in triplicates.

**Table:** 

Gene name	Primer sequence
Refer-actin	Forward 5′ GAGACCTTCAACACCCCAGC 3′ Reverse 5′ ATGTCACGCACGATTTCCC 3′
TGF-β1	Forward 5′ TGCTAATGGTGGACCGCAA 3′ Reverse 5′ CACTGCTTCCCGAATGTCTGA 3′

#### Western blotting of some apoptotic markers

The stored lysed samples were brought to complete protein extraction. The lysate was kept on ice for 30 min in a shaker. Cell debris was removed by centrifugal ion at ∼16,000^x^*g* for 30 min at 4°C. Blots were probed with antibodies against PI3K (phosphatidylinositol 3-kinase)-AKT (serine/threonine-specific protein kinases), Phospho-ERK, Phospho-p38, and Phospho-JNK. The supernatant was transferred to a new tube for further protein concentration determination analysis. Bradford Protein Assay Kit (SK3041) for quantitative protein analysis was provided by BIO BASIC INC. Markham Ontario L3R 8T4 Canada. A Bradford assay was performed according to the manufacturer’s instructions. 20 ug protein concentration of each sample was loaded with an equal volume of 2x Laemmli sample buffer. The components of 2x Laemmli sample loading buffer are as follows: 4%SDS, 10%, mercaptoethanol, 20% glycerol, 0.004% bromophenol blue, 0.125 MTrisHCl. The pH was checked and brought to 6.8. Each of the previous mixtures was boiled at 95°C for 5 min to ensure denaturation of protein before loading on polyacrylamide gel electrophoresis.

#### Histopathological study

At the end of the experiment, skin samples were fixed in 10% neutral buffered formalin at room temperature for 24 h, embedded in paraffin wax, cut into sections 3-4 μm thick, and stained with hematoxylin and eosin (H&E) for general histopathology or Masson’s trichrome (MTC) to detect collagen fiber, as previously mentioned [53]. To quantify the changes, mean epithelial thickness (μm/epithelium) and mean numbers of inflammatory cells infiltrated in the dermis (cells/mm^2^ of the dermis) were determined in general histomorphometric analysis using ImageJ 1.45 software (National Institute of Health, USA) under H&E staining and in collagen fiber-occupied regions of the dermis (area %) under MTC staining.

#### Statistical analysis

Data were analyzed using one-way analysis of variance (ANOVA) followed by the least significant difference principles using SPSS^®^ software. It was expressed as the mean of three experiments ± the standard deviation (SD) or the standard error of the mean (SEM). Statistical differences yielding *P* ˂ 0.0001 were considered significant.

## Results and discussion

### GC/ MS analysis of frankincense oil extracted by hydro-distillation (HD) and microwave-assisted hydro-distillation (MAHD) methods

Essential oils were yielded by extracting frankincense using both extraction methods. MAHD gave a higher oil yield (1.32% vs. 1.1% in HD) at a significantly shorter extraction time (60 min. vs. 4 h in HD). This is in accordance with literature data indicating the acceleration of extractions using microwaves [20]. GC/MS analysis of frankincense oils revealed a total of nineteen identified components, constituting 96.21% and 98.58% of the total essential oils, obtained by HD and MAHD, respectively **(Table 1)**. The major oil component extracted by the two methods was *n*-octyl acetate (75.92% in HD, 80.45% in MAHD). In MAHD extracted oil, other components were α-pinene (3.47%), limonene (2.95%), *n*-octanol (2.27%), verticilla 4(20) 7,11-triene (1.79%) and incensole acetate (1.7%). Other major components extracted by HD were *cis*-β-ocimene (4.23%), α-pinene (2.89%), *trans*-β-ocimene (2.77%), limonene (2.62%), *n*-octanol (1.27%) and linalool (1.75%). Furthermore, *cis*-β-ocimene, sabinene, δ-3-carene, geranyl acetate, camphene and γ-terpinene were detected only by HD, whereas, 1,8 cineol, *cis*-verbenol, *trans*-pinocarveol, cembrene A, cembrene C and incensole were only present in MAHD extracted oil. In both methods, the essential oil was mainly composed of oxygenated compounds, whereas monoterpene and diterpene hydrocarbons constituted only a low percentage of the total extracted oils. It was obvious that the number of oxygenated compounds (80.1% in HD, 87.72% in MAHD) and diterpenes (0.71% in HD, 5.77% in MAHD) increased by MAHD method, in accordance with previous reports [18, 20]. This may be partly due to the fact that microwaves selectively interact with more polar compounds, which are thus more selectively extracted than less polar ones [18, 19]. The predominance of *n*-octyl acetate along with other marker compounds including octanol and diterpene components (verticilla 4(20) 7, 11-triene and incensole acetate) is in line with previous reports on *Boswelia papyrifera* oleogum resin essential oil [8, 54]. In a previous report, MAHD of frankincense (*Boswelia serrata*) essential oil, showed enhanced contents of its main components (α-thujene and α-pinene) [22]. Similarly, the MAHD-extracted frankincense oil (*Boswelia papyrifera*) studied herein revealed increased contents of its above-mentioned marker compounds.

**Table 1:** GC-MS analysis of frankincense oil extracted by hydro-distillation (HD) and microwave assisted hydro-distillation (MAHD) methods.

Retention Index		Compound	Class	Relative %	
**RI** ^**a**^	**RI** ^**L**^			**HD**	**MAHD**
926	924	α-Thujene	Mh	0.38	0.17
937	932	α-Pinene	Mh	2.89	3.47
955	946	Camphene	Mh	0.18	-
975	969	Sabinene	Mh	0.78	-
982	974	β-Pinene	Mh	0.26	0.18
988	988	Myrcene	Mh	0.94	0.16
1012	1008	δ-3-Carene	Mh	0.59	-
1035	1024	Limonene	Mh	2.62	2.95
1033	1026	1,8-Cineole	Om	-	0.15
1038	1032	*cis*-β-Ocimene	Mh	4.23	-
1049	1044	*trans*-β-Ocimene	Mh	2.77	0.64
1064	1059	γ-Terpinene	Mh	0.11	-
1072	1063	*n*-Octanol	Aa	1.27	2.27
1100	1095	Linalool	Om	1.75	1.03
1151	1135	*trans*-Pinocarveol	Om	-	0.31
1154	1137	*cis*-Verbenol	Om	-	0.62
1213	1211	*n*-Octyl acetate	Ae	75.92	80.45
1297	1284	Bornyl acetate	Om	0.25	0.24
1365	1350	Citronellyl acetate	Om	0.09	0.17
1400	1379	Geranyl acetate	Om	0.47	-
1976	1959	Cembrene A	Dh	-	0.99
2015	2002	Cembrene C	Dh	-	0.51
2031	2004	Verticilla-4(20),7,11 triene	Dh	0.36	1.79
2175	2150	Incensole	Od	-	0.78
2197	2189	Incensole acetate	Od	0.35	1.7
Monoterpene hydrocarbons			Mh	15.75	7.57
Diterpene hydrocarbons			Dh	0.36	3.29
**Total Hydrocarbons**			TH	16.11	10.86
Aliphatic alcohols and esters			Aa, Ae	77.19	82.72
Oxygenated monoterpenes			Om	2.56	2.52
Oxygenated diterpenes			Od	0.35	2.48
**Total Oxygenated Compounds**			TO	80.1	87.72
**Total Identified compounds**			TI	96.21	98.58

^a^ Retention index relative to n-alkane on DB5 column.

^L^ Retention index from literature [42] and [55].

Mh: monoterpene hydrocarbon, Dh: diterpene hydrocarbon, aliphatic alcohols: Aa, aliphatic esters: Ae, oxygenated monoterpenes: Om, oxygenated diterpenes: Od.

### *In vitro* anti-collagenase and anti-elastase activities of frankincense oil extracted by HD and MAHD methods

Collagen and elastin are extracellular matrix proteins of the dermal connective tissue, responsible for the structural and mechanical integrity of the skin as well as its elasticity. Their degradation *via* collagenase (class of matrix-degrading metalloproteinases; MMPs) and elastase leads to skin aging [56]. Anti-collagenase and anti-elastase activities have been used as means for estimating the anti-skin aging potential of different natural products and essential oils [57, 58].

In the current study, we analyzed the anti-collagenase and anti-elastase activities of frankincense oils extracted by HD and MAHD methods as a preliminary step to screen their anti-skin aging properties. HD-extracted oil revealed superior anti-collagenase and anti-elastase activities to MAHD-extracted one (IC_50_ 50.9 ± 1.9 and 32.7 ± 0.79 μg/ml in HD-extracted oil vs. 80.7±0.85 and 67.3±0.94 μg/ml in MAHD-extracted oil, for collagenase and elastase, respectively), comparable to epigallocatechin gallate (IC_50_ 24.7 ± 0.87 and 18.2 ± 1.3 μg/ml for collagenase and elastase, respectively). The inhibitory activities of α-pinene and limonene, on both collagenase and elastase enzymes, have been previously proved, whereas a mixture of both exhibited higher anti-collagenase activity than individual compounds indicating synergism. On the contrary, linalool exhibited no activity against both enzymes [59]. β-Ocimene was the main volatile observed to markedly increase in HD vs. MAHD (7% in HD vs. 0.64% in MAHD). In this context, calendula essential oil-loaded vesicular cream, containing *trans* β-ocimene (48.28%) as its major constituent exhibited significant anti-collagenase and anti-elastase activities [60]. It was revealed that the anti-skin aging activity of essential oils did not arise from their major components, yet the synergistic effect of several compounds might contribute to their activities [58]. Thus, the activity of frankincense oil extracted by HD may be attributed to the presence of the aforementioned compounds, however, the synergistic action of these compounds with each other as well as minor components should be considered.

### Preparation of frankincense oil-loaded solid lipid nanoparticles (FO-SLNs) and analysis of the factorial design

#### Preparation of frankincense oil-loaded solid lipid nanoparticles (FO-SLNs)

As per the superior anti-collagenase and anti-elastase activities of frankincense essential oil obtained using the hydro-distillation method (FO), this oil was proposed to be incorporated in solid lipid nanoparticles to improve its solubility, skin permeation and bioavailability before *in vivo* testing. The oily phase consisted of FO and Compritol^®^ 888 ATO as a stabilizer, while the aqueous phase comprised Tween^®^ 80 as a surfactant in distilled water. The planning and the statistical analysis of the general 3^2^ factorial design were completed through Design Expert^®^ software. The percentages of FO (A) and Tween^®^ 80 (B) in the formulation were set as independent variables, whereas PS, ZP and EE% represented the dependent variables. Upon the analysis of the design, the adequate precision ratio values were found to be above 4 and the difference between the predicted R^2^ values and the adjusted R^2^ values was less than 0.2, in all measured responses.

**Fig 1:**
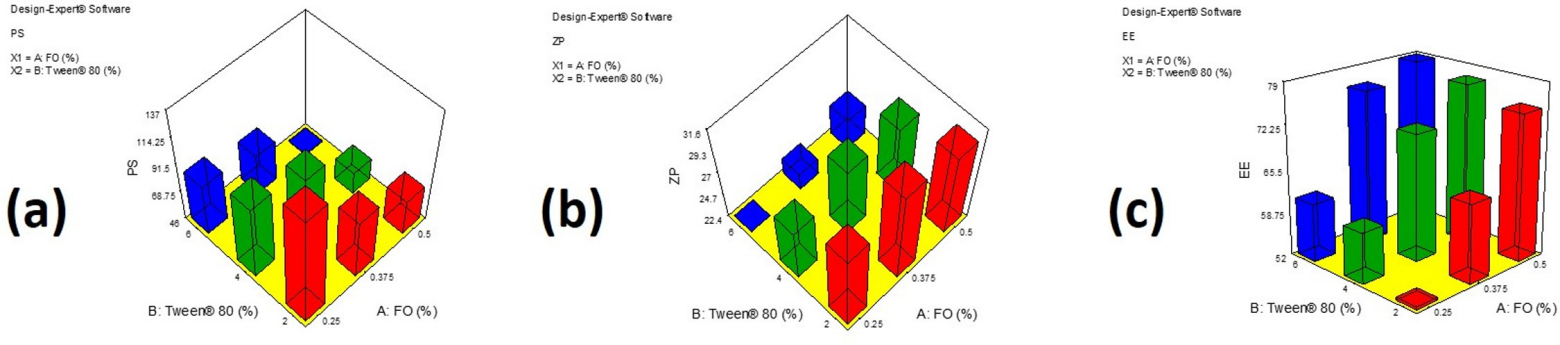
Response 3-D surface plots demonstrating the effect of the independent variables; the percentage of FO in the formulation (A) and the percentage of Tween^®^ 80 in the formulation (B) on the studied variables; (a) Particle size (PS, nm), (b) Zeta potential (ZP, mV) and (c) Entrapment efficiency percentage (EE%).

The PS of the prepared FO-SLNs, represented in **Table 2**, ranged from 46.50±2.12 to 136.50±7.78 nm. The studied independent variables: namely, the percentage of FO (A) and the percentage of Tween^®^ 80 (B) in the formulation affected the PS significantly (*P <* 0.05), as represented in the 3D surface plot **(Fig 1A)**. Higher percentages of FO resulted in decreased PS which might be due to the composition of FO which contains some compounds with solvent-like effects such as limonene. Comparable outcomes were mentioned by Jores *et al*. [61]. Increasing the percentage of Tween^®^ 80 (Hydrophilic lipophilic balance value = 15) [62] resulted in the formation of smaller particles. This could be attributed to the reduction in interfacial tension between two phases which retard the agglomeration of particles [63]. Comparable outcomes were stated earlier by Shaveta *et al*. [64]. It should be mentioned that the results of PDI designate the homogeneity of the size distribution of particles, as PDI values up to 0.5 indicate homogeneously dispersed systems, while that more than this value represents an inhomogeneous system. The prepared FO-SLNs showed PDI values less than 0.5, which indicated the homogeneity of particle size distribution.

**Table 2:** Composition of the prepared FO-SLNs (un-coded units) and their observed responses.

Formulae	Factors		Responses		
	A: FO %	B: Tween^®^80%	Y_I_: PS	Y_2_: ZP	Y_3_: EE %
**F1**	0.25	2	136.50±7.78	-30.40±0.14	52.47±1.06
**F2**	0.25	4	106.50±9.19	-27.70±0.14	59.90±1.56
**F3**	0.25	6	88.00±5.66	-22.40±0.42	61.12±1.70
**F4**	0.375	2	94.95±1.34	-31.55±0.35	64.23±0.77
**F5**	0.375	4	94.00±0.00	-29.10±0.14	71.95±0.09
**F6**	0.375	6	79.00±2.83	-24.05±0.07	76.22±1.13
**F7**	0.5	2	74.945±4.32	-30.45±0.92	75.03±0.67
**F8**	0.5	4	64.00±5.66	-28.70±0.42	77.24±0.42
**F9**	0.5	6	46.50±2.12	-25.30±0.28	78.83±1.34

FO, Hydro-distilled frankincense oil; PS, Particle size; ZP, Zeta potential; EE %, Entrapment efficiency percentage.

The ZP values of FO-SLNs ranged between -22.40±0.42 and -30.40±0.14 **(Table 2)**. These high negative ZP values warranted the formation of physically stable systems [65]. The studied independent variables: namely, the percentage of FO (A) in the formulation and the percentage of Tween^®^ 80 (B) in the formulation affected the ZP significantly (*P <* 0.05), as represented in the 3D surface plot **(Fig 1B)**. Incorporating higher percentages of FO led to the formation of SLNs with higher ZP (absolute) values due to the presence of oxygenated components in the oil. Moreover, a higher percentage of Tween^®^ 80 resulted in particles with higher ZP values (absolute) due to the stabilizing effect of the surfactant which guarded against the agglomeration of the particles and ensured the electric repulsion between particles [30]; moreover, this was conjugated with smaller PS. Xiong *et al*. stated that in the presence of Tween 80 in the solution, zeta potential values became more negative [66].

The calculated EE % was found to be in the range of 52.47±1.06 to 78.83±1.34% as shown in **Table 2**. The studied independent variables: namely, the percentage of FO (A) in the formulation and the percentage of Tween^®^ 80 (B) in the formulation affected the EE % significantly (*P <* 0.05), as represented in the 3D surface plot **(Fig 1C)**. It goes without saying that higher EE % were observed with elevated percentages of FO. Increased percentages of Tween^®^ 80 assisted the solubilization of more amounts of oil in the nanosystems which were proved to have higher EE%.

#### Selecting the optimal FO-SLNs

The optimal FO-SLNs was nominated as per the outcomes of the factorial design with minimizing PS and maximizing ZP (absolute value) and EE%. The optimal formulation (F8) prepared with 0.5% FO and 4% Tween^®^ 80, showed a PS of 64.00±5.66, ZP of—28.70±0.42 mV, and EE% of 77.24±0.42%.

#### *In vitro* characterization of the optimal FO-SLNs formulation

**Fig 2:**
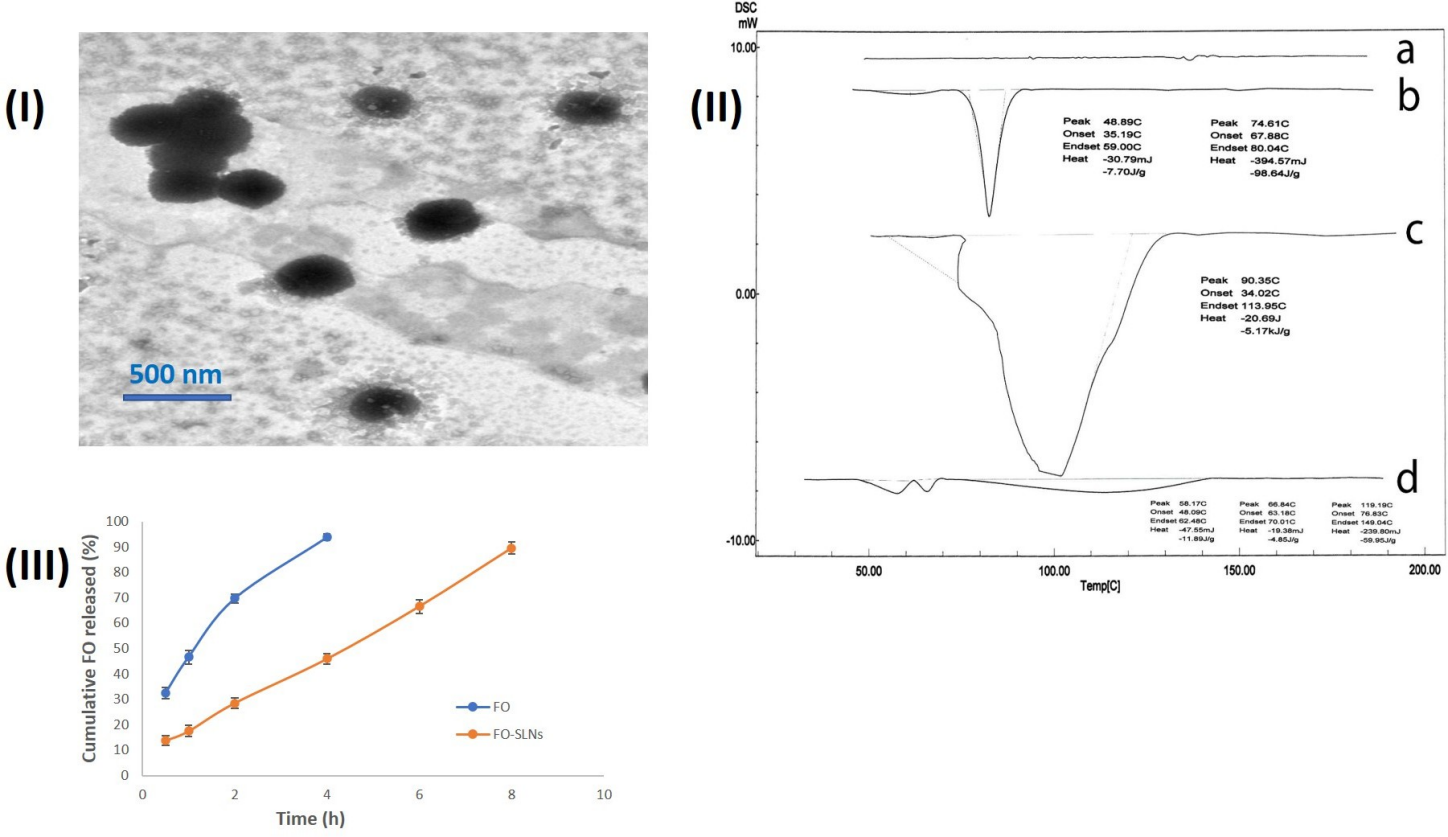
**(I)** TEM micrographs of the optimal FO-SLNs (F8), **(II)** DSC thrmograms of FO (a), Compritol^®^ 888 ATO (b), a physical mixture of FO and Compritol^®^ 888 ATO (c), and the optimal FO-SLNs (F8) (d), and **(III)** Mean % cumulative drug release profiles of FO and FO-SLNs in phosphate buffer (pH 6, simulating skin surface pH) at 32 ± 0.5°C (n = 3).

TEM image of the optimal FO-SLNs formulation (F8) evidently displayed uniform, discrete spherical nanoparticles with sizes comparable to that obtained *via* the Zetasizer **(Fig 2I)**. **Fig 2II** illustrates the DSC thermograms of pure FO, Compritol^®^ 888 ATO, a physical mixture of FO and Compritol^®^ 888 ATO, and F8. The thermogram FO showed no distinct peak, while that of Compritol^®^ 888 ATO displayed a sharp endothermic peak at 74.61°C [67], while that of the physical mixture of FO and Compritol^®^ 888 ATO exhibited the endothermic peak of Compritol^®^ 888 ATO. Thermograms of F8 disclosed a wider and less sharp peak of the lipid due to the small size of the particles (nanometer range), and the distributed lipid condition, in addition to the presence of surfactant. The enthalpy and crystallinity of FO-SLNs were lower than those of the initial lipid, indicating an increased number of lattice defects in FO-SLNs [68]. The results of the characteristics of F8 showed substantially increased PS (p *<* 0.05), while the ZP and EE% values were unchanged at 25 ± 2°C/60 ± 5% RH (p *>* 0.05). However, upon kept stored at 5 ± 3°C, F8 revealed an insignificant change in the PS, ZP and EE% values (p *>* 0.05). The obtained results endorsed the storing of F8 at the lower temperature to guarantee unchanged particulate features. The cumulative percentage of FO released over 8 h in buffers simulating the pH of the skin was studied as shown in **Fig 2III**. FO was released completely within 4 hours, while FO was released from the formulation in a slower pace which is strongly related to the results of EE% confirming the successful incorporation of FO in the solid lipid nanoparticles.

### *In vivo* study of the anti-photoaging activity of frankincense essential oil

Based on the promising anti-collagenase and anti-elastase activities of hydro-distilled frankincense oil (FO) proved herein, we hypothesized that FO may possess potential anti-aging properties. Consequently, the efficacies of FO and its designed solid lipid nanoparticle formulation (FO-SLNs) were examined in UVB-irradiated rats.

Skin tissue has the role of a barrier against harmful external factors: pathogens, chemicals, and UV, thus is directly exposed to several diseases and aging. Including harmful agents, UV radiation is the main factor that damages the skin [69] with long-term significance, including photoaging, photoimmunosuppression and photocarcinogenesis [70, 71] *via* inducing ROS production, which activates the expression of MMPs *via* the MAPK and PI3K/Akt signaling pathways, thereby leading to wrinkling of the skin; an evident hallmark of skin photoaging [72]. Therefore, to examine the possible mechanisms by which FO and FO-SLNs can protect against UVB-induced skin damage, the levels of SOD, CAT, IL-6, NF-κB, procollagen I, MMP1, MMP9 along with the expressions of TGF-β1, pERK, pJNK, and pp38 as well as histological analysis were assessed.

**Fig 3:**
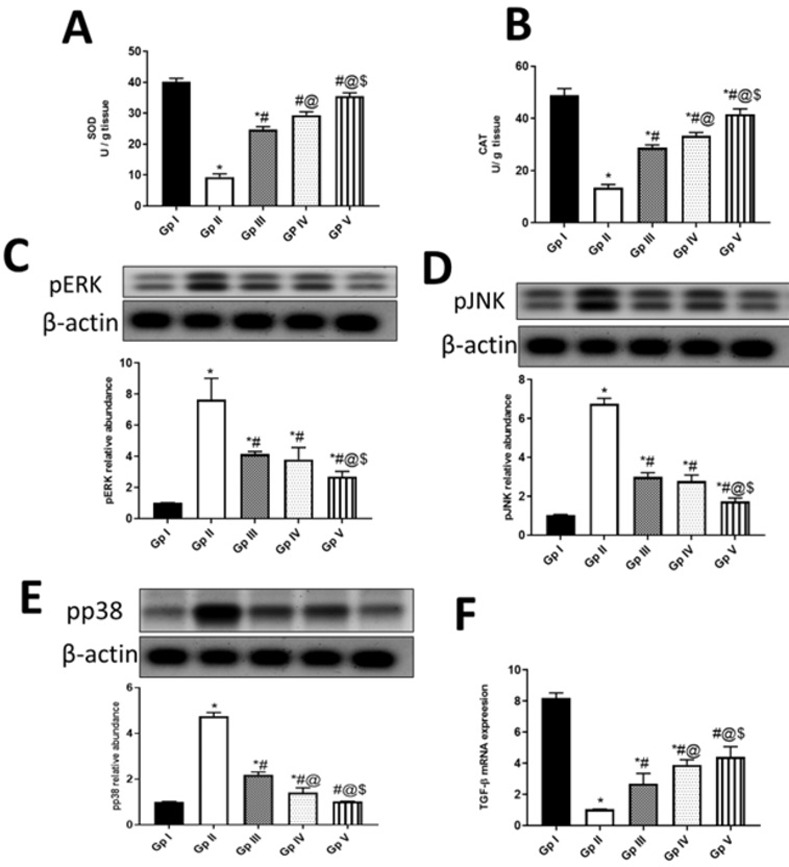
Effect of FO (Gp IV) and FO-SLNs (Gp V) on antioxidant biomarkers, MAPK and TGF-β pathways. **A;** superoxide dismutase (SOD) activity (U/ g tissue), **B;** catalase (CAT) activity (U/ g tissue), C; protein kinase RNA-like endoplasmic reticulum kinase (pERK), D; Phosphorylated Jun N- Terminal Kinase (pJNK), E; phosphorylated p38 (pp38), F; The m-RNA expression of transforming growth factor beta (TGF-β). In comparison with the normal control group (Gp I), UVB group (Gp II) and the positive standard group (Gp III). Data are presented as mean ±SD for 6 rats. *Statistically significant difference from normal control group (p˂0.0001), #statistically significant difference from the UVB group (p˂0.0001), @statistically significant difference from the positive standard group (Vitamin A palmitate) (p˂0.0001), $statistically significant difference from FO (unformulated frankincense oil) group (p˂0.0001).

Skin aging is closely related to the induction of oxidative stress in dermal fibroblasts [73], and so drugs that could halt oxidative damage in dermal fibroblasts are attracting candidates for skin aging in the cosmeceutical industry. In that regard, SOD and CAT, endogenous antioxidant defense enzymes, are critical in oxidative stress. SOD is crucial for superoxide free radicals removal through the oxidative stress condition, leading to the production of H_2_O_2_ which is in turn eradicated by CAT and other peroxidases [74]. In the current study, UVB-induced oxidative stress was reflected in significantly decreased levels of SOD (9.4 ± 0.9859 U/ g tissue) and CAT (13.45 ± 1.26 U/g tissue) in homogenized skin of UVB-irradiated rats, compared to the normal control group (SOD: 40 ± 1.095 U/g tissue, CAT: 48.85 ± 2.574 U/g tissue) **(Fig 3A and 3B)**. Frankincense oil significantly increased SOD and CAT levels in FO (by 212.8 and 147.6%, respectively) and FO-SLNs (by 277.7 and 209.7%, respectively) groups at *P* ˂ 0.0001, revealing more significant effects than the positive standard; vitamin A palmitate (163.3 and 114.5%, respectively). So, the proposed treatments enhanced the level of H_2_O_2_-detoxifying enzyme, CAT alongside the ROS removal enzyme, SOD, promoting the capacity of fibroblasts to eliminate harmful ROS, suppressing oxidative damage, and preventing cell death. FO-SLNs group was superior to the FO group, indicating the ability of the formulation to increase the solubility of the oil, and hence its penetration potentiality.

In good agreement, the incorporation of commercial frankincense oil (with no given chemical characterization) enhanced the DPPH radical scavenging activity of psyllium-carbopol/frankincense oil hydrogel wound dressing [17]. Additionally, frankincense essential oil (*B*. *carterii*, enriched in α-pinene) prompted cancer cell death in human bladder cancer cells through NRF-2-mediated oxidative stress [75]. Among the major constituents identified herein in FO, α-pinene, limonene, linalool and β-ocimene may contribute to its antioxidant properties [60, 76–78].

**Fig 4:**
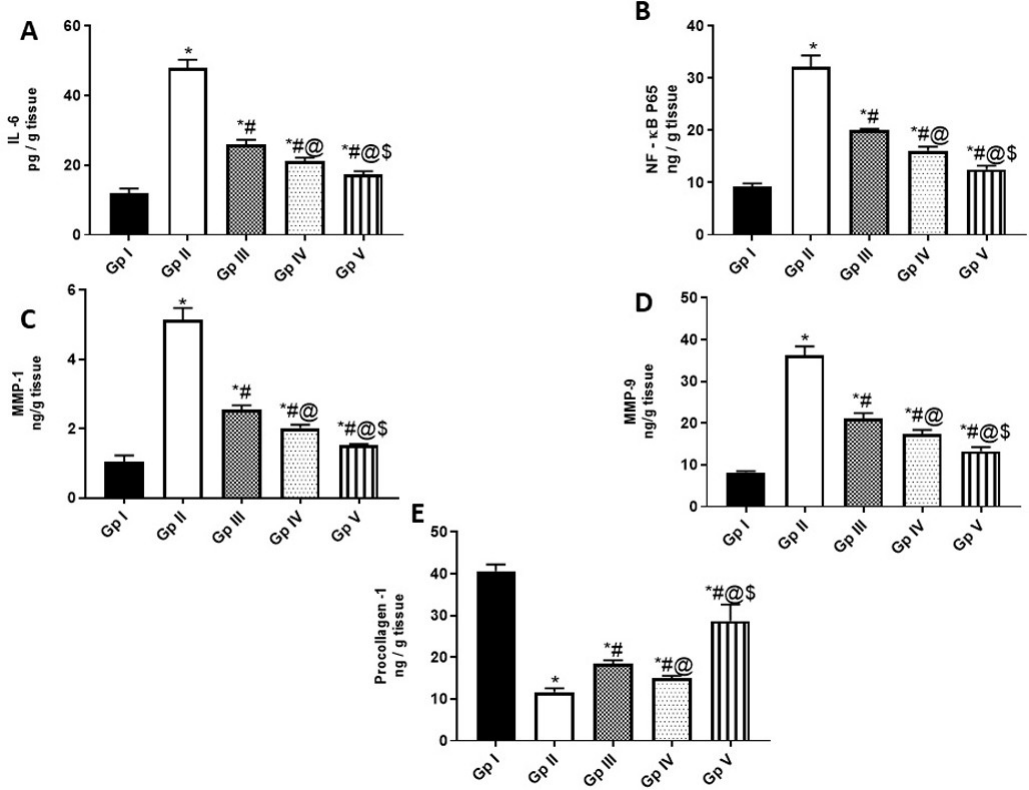
Anti-inflammatory and anti-aging activities of FO (Gp IV) and FO-SLNs (Gp V). **A;** The levels of Interleukin (IL)-6 (pg/ g tissue), **B;** The levels of nuclear factor (NF)-κBP65 (ng/ g tissue), **C;** The levels of Matrix metalloproteinase (MPP 1) (ng/ g tissue), **D;** The levels of Matrix metalloproteinase 9 (MPP 9) (ng/ g tissue), **E;** The levels of procollagen 1(ng/ g tissue). In comparison with the normal control group (Gp I), UVB group (Gp II) and the positive standard group (Gp III). Data are presented as mean ±SD for 6 rats. *Statistically significant difference from normal control group (p˂0.0001), #statistically significant difference from the UVB group (p˂0.0001), @statistically significant difference from the positive standard group (Vitamin A palmitate) (p˂0.0001), $statistically significant difference from FO (unformulated frankincense oil) group (p˂0.0001).

UVB-induced photoaging is initiated by the production of ROS, that in turn activates several receptors for interleukins and transforming growth factors. Stimulation of these receptors induces downstream signaling pathways of mitogen-activated protein kinases (MAPKs) such as pp38, c-Jun amino-terminal kinase (JNK), and extracellular signal-regulated kinases (ERK), leading to activation of nuclear transcription factors such as NF-κB [79]. NF-κB plays a vital role in the inflammatory signaling cascade, inducing the expression of proinflammatory cytokines such as IL-6, which in turn activate NF-κB in an endless cycle. Additionally, NF-κB upregulates MMPs expressions, which play a key role in connective tissue damage due to increased collagen degradation [80]. On the other hand, the transforming growth factor-beta 1 (TGF-β1) is acclaimed to be the major regulator of procollagen type I synthesis [81]. In our study, UVB radiation-induced the expressions of pERK, pJNK and pp38 (7.65 ± 1.369, 6.76 ± 0.2848 and 4.75 ± 0.1643, respectively) **(Fig 3C–3E)** and downregulated mRNA expression of TGF-β1 **(Fig 3F)** (1.045 ± 0.016) in UVB-irradiated rat skin, compared to the normal control group (1.02 ± 0.01095, 1.045 ± 0.02739, 1.015 ± 0.005477 and 8.2 ±0.329, respectively). Frankincense oil in both FO and FO-SLNs groups as well as the positive standard downregulated UVB-induced expressions of pERK, pJNK and pp38 (by 50.3,58.8 and 75% for FO; 64.6, 74.1 and 78.4% for FO-SLNs; 45.7, 55.6 and 53.9% for positive standard, respectively), and upregulated TGF-β (by 273.2, 322 and 157.9%, respectively) in UV-injured rats. It is worthy to document the superior effect of FO-SLNs in inhibiting UVB-induced MAPK signaling pathway and upregulating TGF-β expression, compared to FO and positive standard groups. In consequence, significantly enhanced levels of inflammatory markers (IL-6 and NF-κB) were evidenced in UVB-irradiated rats’ skin (47.85 ± 2.465 pg/ g tissue and 32.2 ± 2.191 ng/ g tissue), **(Fig 4A and 4B)** indicating inflammation, compared to the normal control group (11.9 ± 1.424 pg/ g tissue and 9.25 ±0.6025 ng/ g tissue). FO, FO-SLNs and positive standard groups significantly inhibited UVB-induced inflammatory response (by 55.7 and 50% for FO; 63.7 and 61% for FO-SNs; 45.9 and 37.7% for the positive standard group, respectively), whereas FO-SLNs was the most significantly potent treatment. In accordance, the topical anti-inflammatory effect of frankincense essential oil (*Boswellia* spp., rich in octyl acetate and octanol), mediated via downregulating COX-2, IL-6, TNF-α and NF-κB, has been demonstrated in TPA-induced mouse ear edema model [16]. Interestingly, the incorporation of commercial frankincense oil (with no given chemical characterization) enhanced the anti-inflammatory (IL-6 and NF-κB) effect of Psyllium-Carbopol hydrogel wound dressing in a rat model [17]. The anti-inflammatory effect of FO proven here may be attributed to α-pinene, limonene, linalool and 1-octanol, among its major constituents reported in the current study [13, 76–78, 82, 83].

In the skin, MMP1, MPP9 and other proteases induce the degradation of collagen I, rendering them attractive targets for anti-photoaging agents. Chronic UVB exposure was found to reduce the production of procollagen type I, causing collagen loss, skin thickening, increased wrinkling, and decreased skin elasticity [81]. So, MMP1, MMP9 and pro-collagen parameters were assessed to investigate the protective effect of our treatments (FO & FO-SLNs) against UVB-induced aging and wrinkles **(Fig 4)**. MMP1 and MMP9 levels significantly increased (5.135 ± 0.3451 & 36.35 ± 2.027 ng/g tissue, respectively) in UVB-injured rat skin, whereas procollagen I levels showed a significant decline (11.65 ± 0.9311 ng/ g tissue), compared to the normal control group (1.05 ± 0.1753, 8.2 ± 0.3286 and 40.65 ± 1.588 ng/ g tissue, respectively) **(Fig 4C–4E)**. Interestingly, frankincense oil in both groups (FO and FO-SLNs) attenuated the induced- UVB irradiation levels of collagen regulatory factors (MMP1, MMP9 and procollagen I), revealing more remarkable anti-aging effects, compared to the positive standard; vitamin A palmitate, with FO-SLNs as the best treatment. Thus, among the mechanistic ways by which frankincense oil can exert its anti-photoaging effect; are upregulation of TGF-β1 expression which induced procollagen type I synthesis and inhibition of MAPK pathway, thereby NF-κB and MMPs levels.

In line, the promising potential of frankincense oil (enriched in α-pinene) in tissue remodeling and immunomodulation was previously reported in human fibroblasts [10]. Alongside, the incorporation of commercial frankincense oil (with no given chemical characterization) enhanced MMP-9 and wound healing efficiencies of Psyllium-Carbopol hydrogel wound dressing in a rat model [17]. In that regard, among the major constituents of FO investigated herein, α-pinene and linalool protected against skin photoaging in UVA-irradiated mice and UVB-irradiated human skin cells, respectively *via* inhibition of MMPs expression (MMP-9 and MMP-2) through prevention of NF-κB activation and the consequent inhibition of inflammatory cytokines (TNF-α, IL-6), together with preventing oxidative stress-activated protein kinases [76, 77, 83]. Linalool also prevented induced hyperplasia and degradation of collagen in chronic UVB-exposed mice skin, thus is considered an effective photo-chemo-preventive agent [84], whereas α-pinene histologically inhibited damage of dermal tissue in UVA-irradiated mice [77]. To note, limonene, another FO major constituent, accelerated skin wound healing in rats, showing a significant increase in re-epithelialization, granulation tissue thickness and angiogenesis [85], as well d-limonene reduced matrix metalloproteinases expression (MMP-1 and MMP-13) together with inhibiting nitric oxide (NO) production, NF-κB and protein kinases (JNK and p38) activation in an osteoarthritis cell model [78]. Nevertheless, [60] reported the effectiveness of calendula essential oil-loaded vesicular cream (containing trans-Β-ocimene as a major constituent) against skin aging *via* its high in vitro sun protection factor and remarkable antioxidant capacity.

**Fig 5:**
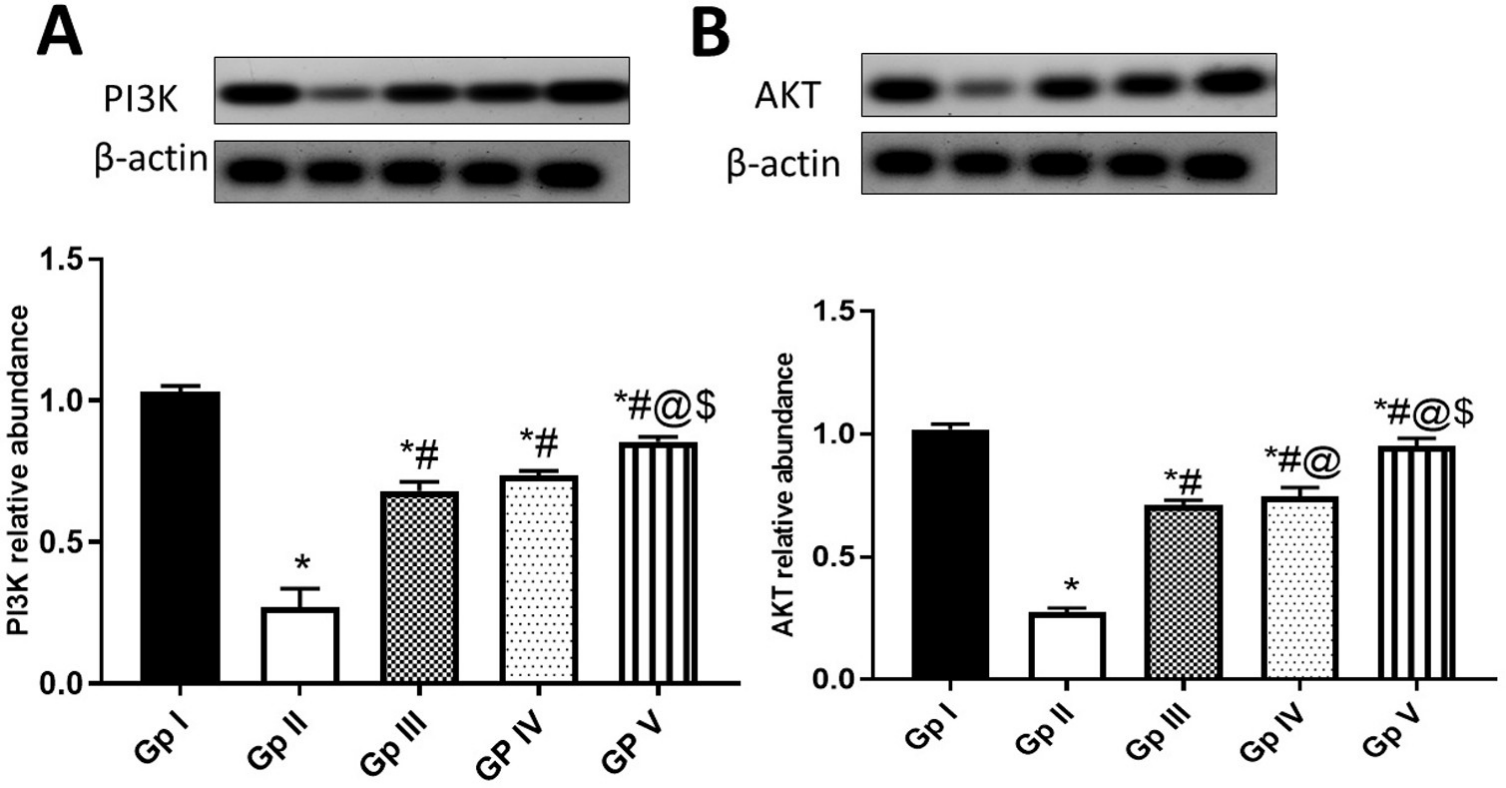
Effect of FO (Gp IV) and FO-SLNs (Gp V) on PI3K/AKT pathway. A; Phosphoinositide 3-kinases (PI3K), B; alpha serine/threonine-protein kinase (AKT). In comparison with the normal control group (Gp I), UVB group (Gp II) and the positive standard group (Gp III). Data are presented as mean ±SD for 6 rats. *Statistically significant difference from normal control group (p˂0.0001), #statistically significant difference from the UVB group (p˂0.0001), @statistically significant difference from the positive standard group (Vitamin A palmitate) (p˂0.0001), $statistically significant difference from FO (unformulated frankincense oil) group (p˂0.0001).

Furthermore, the PI3K (phosphatidylinositol 3-kinase)- AKT pathway is one of the most important signalings in melanoma, thereby PI3K/AKT signaling inhibitors have attracted more attention in recent decades [86]. In this study, UVB exposure decreased PI3K **(Fig 5A)** and AKT **(Fig 5B)** expressions in UVB-injured rat skin (0.27 ± 0.06573 & 0.275 ± 0.01643), compared to the normal control group (1.03 ± 0.02191 & 1.02 ± 0.02191). The induction levels of PI3K/AKT within FO treatments were documented here **(Fig 5A and 5B),** where FO-SLNs group was the most efficacious treatment, followed by FO and positive standard groups. This is in line with the previously reported anti-proliferative activity of frankincense oil (enriched in α-pinene) in pre-inflamed human dermal fibroblasts [10], which can be related in our study to FO major identified constituents viz., α-pinene, limonene and linalool [77, 84, 87, 88].

**Fig 6:**
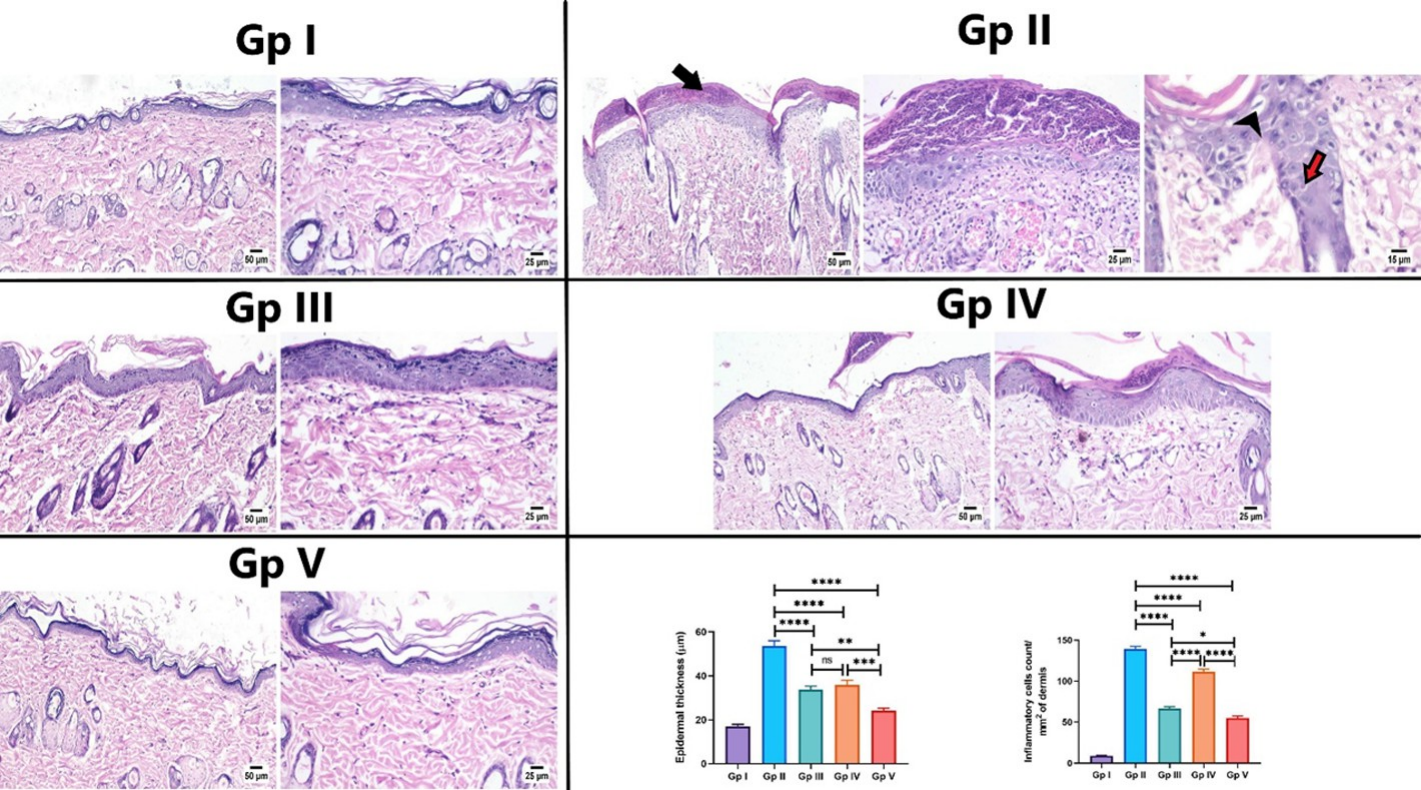
Photomicrograph of skin sections of different experimental groups (H&E). Gp I (normal control group) showed normal thin epidermal layer and healthy dermal collagen fibers. Gp II (UVB group) showed thick serocellular crust covering the epidermis (arrow), with congested blood capillaries and inflammatory cells infiltration in the dermal layer, note hydropic degeneration (arrowhead) of epithelial cells with presence of sunburn cells (red arrow). Gp III (positive standard group) showed moderate thickened epidermal layer with fewer number of inflammatory cells infiltrating the dermal layer. Gp IV (FO group) showed thin crust covering with moderate thickened epidermis. Gp V (FO-SLNs group) showed apparently normal histological structure of epidermis and dermis. Charts showing epidermal thickness and inflammatory cells infiltration in the dermal layer in different groups. Data are presented as mean ±SE. Significant difference was considered at p<0.05.

The previously investigated biochemical parameters in the present study were further confirmed by histological examination of rat skin in different experimental groups. Histological examination of the normal control group revealed a normal histological structure of skin sections, characterized by a normal epidermis composed of stratified squamous keratinized epithelium, accompanied by normal dermis consisting of loose connective tissue containing hair follicles and associated sebaceous glands **(Fig 6).** In UVB irradiated group, rats showed several histopathological alterations in the examined tissue sections. The epidermis was covered by a thick necrotic serocellular crust. The epidermal layer was markedly thickened with an increased number of surface microfolds, accompanied by spongiosis and hydropic degeneration of the epithelial cells with the existence of sunburn cells. A focal ulcerated area was noticed, in some sections, that was covered by karyorrhectic, eosinophilic tissue debris and bacterial clusters. The dermis exhibited dermatitis that was more prominent in the superficial dermal layer. The dermal collagen bundles were dispersed by edema, congested blood vessels and hemorrhages in some circumstances. Numerous numbers of inflammatory cells were infiltrated in the affected dermal tissue **(Fig 6).**

The positive standard group (Vitamin A palmitate) showed marked protection of skin tissue except for a few alterations. The number of microfolds was moderately increased, compared to the normal control group. Epidermal thickness was increased in some examined sections and appeared normal in other sections. The micrographs showed the infiltration of the dermis with a limited number of inflammatory cells in fewer areas with apparently normal histological structure in most examined sections **(Fig 6).**

As per the results of the FO group, frankincense oil showed lower protection against UV irradiation in the exposed skin tissue. The epidermis was of variable thickness with a prominent increase in surface microfolds. Spongiosis and vacuolar degeneration were noticed in some epidermal areas that also showed thin serocellular crust in some sections. The dermis showed moderate dermatitis, characterized by few number of inflammatory cells and erythrocytes infiltration with edema and congested blood capillaries **(Fig 6).**

Application of FO-SLNs exerted the highest protection, where most examined sections showed apparently normal histological structure of the epidermis and dermis **(Fig 6).**

In addition, the histomorphometric analysis of epidermal thickness showed a significant increase in the UV-irradiated group, when compared to other groups. Meanwhile, the absence of a significant difference was recorded between the positive standard and FO groups. The lowest epidermal thickness was detected in the FO-SLNs group, showing a significant decrease in epidermal thickness when compared to other groups **(Fig 6).** Moreover, the number of inflammatory cells infiltration was significantly increased in the dermal layer in the UVB-irradiated group, compared to other experimental groups. Meanwhile, the lowest number of inflammatory cells was recorded in the FO-SLNs group, showing a significant decrease in comparison to the UVB-irradiated group and other treatment groups **(Fig 6).**

**Fig 7:**
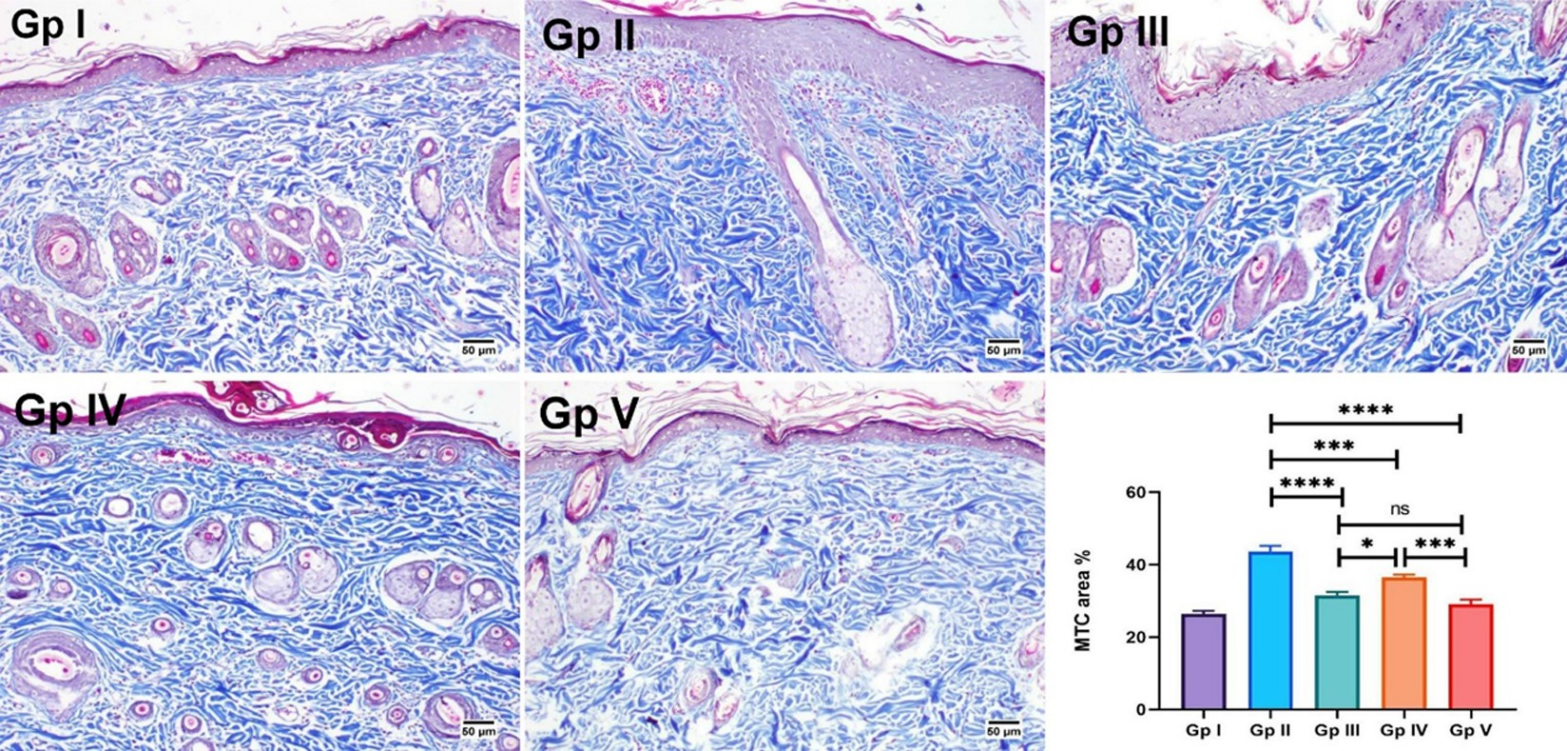
MTC-skin-stained sections for evaluation of collagen fibers. Gp I (normal control group) showed normal collagen bundles. Abnormal accumulation of collagen fibers is found in Gp II (UVB group) and Gp IV (FO group). Apparently normal collagen is shown in Gp V (FO-SLNs group). Chart present the area % of MTC staining in different groups. Data are presented as mean ±SE. Significant difference was considered at p<0.05.

Finally, dermal sclerosis was evaluated in the MTC-stained skin sections of different experimental groups. The normal control group showed normal distribution of collagen fibers in all examined tissue sections. However, abnormal accumulation of collagen bundles was detected in the UVB irradiated group, showing a significant increase in area % expression, compared to other groups. No significant difference was found between the positive standard and FO groups **(Fig 7).** Consequently, SLNs seem a promising nanocarrier for FO to improve its solubility and hence increase skin permeation resulting in better therapeutic outcomes.

## Conclusions

Conclusively, hydro-distilled frankincense oil and its solid lipid nanoparticles prohibited UVB-induced aging and wrinkle formation in rats *via* inhibiting ROS, MAPK (pERK, pJNK, and pp38) and PI3K/AKT signaling pathways as well as NF-κB, alongside upregulating TGF-β pathway. This effect is likely due to the several constituents in FO which could exert additive or synergistic effects. Using histological techniques, we observed the changes associated with photoaging in rats treated with FO, specifically, rats treated with FO-SLNs had thinner epidermal layers and denser collagen fibers compared with untreated ones. To note, the designed FO-SLNs formulation revealed a superior anti-photoaging effect, compared to unformulated oil and positive standard, highlighting the use of SLNs as a promising nanocarrier for FO that improved its solubility and hence increased skin permeation, resulting in better therapeutic outcomes. Our study draws attention to the promising effect of FO-SLNs as a potential anti-aging therapy, necessitating further clinical trials which should now follow.
